# Editorial: Insights in cancer endocrinology: 2022

**DOI:** 10.3389/fendo.2023.1236887

**Published:** 2023-07-07

**Authors:** Claire M. Perks, Antonino Belfiore

**Affiliations:** ^1^Bristol Medical School, Department of Translational Health Sciences, University of Bristol, Bristol, United Kingdom; ^2^Department of Clinical and Experimental Medicine, University of Catania, Catania, Italy

**Keywords:** endocrine malignancies, medullary thyroid cancer, rare endocrine malignancies, neuroendocrine neoplasms (NENs), parathyroid carcinoma

It is our pleasure to introduce this Research Topic entitled “*Insights in cancer endocrinology: 2022*” that particularly highlights some of the issues surrounding rarer endocrine malignancies, such as parathyroid carcinoma (PC) and medullary thyroid (MT) cancer.

Neuroendocrine neoplasms (NENs) can occur in different locations in the body, for example the pancreas and lung. They are composed of two types: well-differentiated and low-proliferating NENs - neuroendocrine tumours (NETs) and poorly differentiated, highly proliferating NENs- neuroendocrine carcinomas (NECs). Puliafito et al. conducted a retrospective study that included 70 patients with lung NECs to compare the outcome data, including disease control rate, overall and progression-free survival for patients treated with either cisplatin or carboplatin.

Thyroid cancer (TC) is the most common endocrine malignancy accounting for 586202 new cases and 43646 deaths in 2020 (1). The identification of four main sub-types of TC, including differentiated, poorly differentiated, medullary, and anaplastic, has led to a better understanding of the molecular progression of the disease. The important contribution of tyrosine kinase inhibitors (TKIs) in the treatment of TC is highlighted in a comprehensive review by Puliafito et al., in which clinical trials demonstrating improved progression-free survival are cited along with a discussion on their associated toxicities and how this may be managed.

Medullary TC is the rarest sub-type accounting for approximately 4% of all TCs (2) and in their overview, Zhang et al. appraise the current guidelines for the management of MTC. Located on or near the thyroid gland are the parathyroid glands. Primary hyperparathyroidism (PHPT) occurs when one or more of these glands over-secrete parathyroid hormone (PTH). Barale et al. focus on two rare causes of PHPT, PC and atypical carcinoma (AC). Their study aimed to identify factors particular to these carcinomas to enable the prediction of the clinical outcome of PC or AC following surgery.

We have enjoyed hosting this exciting Research Topic and we thank all the authors for their excellent contributions.

## Author contributions

CP wrote the editorial. CP and AB commented on a draft and agreed to the final version.
